# *myh9b* is a critical non-muscle myosin II encoding gene that interacts with *myh9a* and *myh10* during zebrafish development in both compensatory and redundant pathways

**DOI:** 10.1093/g3journal/jkae260

**Published:** 2024-11-06

**Authors:** Laura A Rolfs, Elizabeth J Falat, Jennifer H Gutzman

**Affiliations:** Department of Biological Sciences, University of Wisconsin-Milwaukee, Milwaukee, WI 53211, USA; Department of Biological Sciences, University of Wisconsin-Milwaukee, Milwaukee, WI 53211, USA; Department of Biological Sciences, University of Wisconsin-Milwaukee, Milwaukee, WI 53211, USA

**Keywords:** zebrafish, genetics, *MYH9*, *MYH10*, *myh9a*, *myh9b*, *myh10*, zebrafish development, non-muscle myosin, NMIIA, NMIIB

## Abstract

Non-muscle myosin (NMII) motor proteins have diverse developmental functions due to their roles in cell shape changes, cell migration, and cell adhesion. Zebrafish are an ideal vertebrate model system to study the NMII encoding *myh* genes and proteins due to high sequence homology, established gene editing tools, and rapid ex utero development. In humans, mutations in the NMII encoding *MYH* genes can lead to abnormal developmental processes and disease. This study utilized zebrafish *myh9a*, *myh9b*, and *myh10* null mutants to examine potential genetic interactions and roles for each gene in development. It was determined that the *myh9b* gene is the most critical NMII encoding gene, as *myh9b* mutants develop pericardial edema and have a partially penetrant lethal phenotype, which was not observed in the other *myh* mutants. This study also established that genetic interactions occur between the zebrafish *myh9a*, *myh9b*, and *myh10* genes where *myh9b* is required for the expression of both *myh9a* and *myh10*, and *myh10* is required for the expression of *myh9b*. Additionally, protein analyses suggested that enhanced NMII protein stability in some mutant backgrounds may play a role in compensation. Finally, double mutant studies revealed different and more severe phenotypes at earlier time points than single mutants, suggesting roles for tissue specific genetic redundancy, and in some genotypes, haploinsufficiency. These mutants are the first in vivo models allowing for the study of complete loss of the NMIIA and NMIIB proteins, establishing them as valuable tools to elucidate the role of NMII encoding *myh* genes in development and disease.

## Introduction

Non-muscle myosin II (NMII) proteins are highly conserved molecular motor proteins that modulate force generation within cells through their interaction with the actin cytoskeleton. Through their critical roles in regulating the actin cytoskeleton, NMII proteins participate in diverse cellular processes such as: cell shape changes, cell division, cell migration and cancer, polarity, mechanotransduction, and adhesion (Swailes *et al*. 2006; Beadle *et al*. 2008; Choi *et al*. 2008; Gutzman and Sive 2010; Ouderkirk and Krendel 2014; Gutzman *et al*. 2015; Sahu *et al*. 2017; Popa and Gutzman 2018; Halder *et al*. 2021). NMII proteins function as hexameric structures, consisting of two heavy chains (encoded by the *MYH* genes), two essential light chains, and two regulatory light chains (Dulyaninova and Bresnick 2013). The regulatory light chains are phosphorylated through upstream signaling pathways to regulate NMII activity (Garrido-Casado *et al*. 2021). Mammals express three NMII heavy chain isoforms, each encoded by distinct *MYH* genes. These isoforms include NMIIA (*MYH9*), NMIIB (*MYH10*), and NMIIC (*MYH14*) (Simons *et al*. 1991; Golomb *et al*. 2004).

In humans, mutations in the NMII encoding *MYH* genes can result in disease, confirming their essential roles in cells. *MYH9* gene mutations may result in *MYH9-*related disease (*MYH9*-RD), which is characterized by symptoms including macrothrombocytopenia, leukocyte inclusion bodies, nephritis, visual defects, and hearing loss (Kelley *et al*. 2000; Lalwani *et al*. 2000; Seri *et al*. 2000; Kunishima *et al*. 2001; Seri *et al*. 2002, 2003). While mutations in the *MYH10* gene have not been associated with any known human genetic disorders, two human patients with de novo *MYH10* mutations presented with clinical phenotypes including microcephaly, congenital diaphragmatic hernia, respiratory failure, and cardiac abnormalities, among others (Tuzovic *et al*. 2013; Gardner *et al*. 2020). Finally, *MYH14* gene mutations have been associated with the development of hearing loss (Donaudy *et al*. 2004; Kim *et al*. 2017).

Due to their implications in human disease, the generation of in vivo models is essential to identify the roles of the NMII encoding genes during development. Mouse models with patient-specific and highly conserved mutations in the homologous sequence of the *Myh9* and *Myh10* genes have advanced the understanding of genotype-phenotype disease development (Ma *et al*. 2004; Zhang *et al*. 2012; Suzuki *et al*. 2013). However, complete ablation of NMIIA in mice is embryonic lethal, as it is required for the formation of cell–cell adhesions and the development of a polarized visceral endoderm (Conti *et al*. 2004), and complete ablation of NMIIB in mice is lethal before or within the first day of life as it is required for normal cardiac development (Tullio *et al*. 1997). Therefore, to identify the roles of *myh9* and *myh10* in developmental processes, these studies utilized the zebrafish (*Danio rerio*) as a model organism. The zebrafish model is ideal to study the NMII encoding *myh* genes during development as they share high gene and protein homology with humans, have well established genome editing techniques, and develop rapidly ex utero allowing for the examination of early developmental processes.

We hypothesized that null zebrafish mutants would provide the models needed to identify the role of these genes, and the possible genetic interactions between them, during vertebrate development. In addition, we postulated that zebrafish mutant studies would fill a gap in our knowledge of the role of NMII proteins in development, which has not been previously examined due to the lethality in mouse models. Zebrafish contain four NMII encoding *myh* genes: *myh9a*, *myh9b*, *myh10*, and *myh14*. This study examined the *myh9a*, *myh9b*, and *myh10* genes, but not *myh14* since *Myh14* was not found to be essential for mammalian development (Ma *et al*. 2010), and has lower sequence homology between zebrafish and human. To test our hypothesis, we obtained a *myh9a* null zebrafish mutant line from the Sanger Zebrafish Mutation Project and we generated *myh9b* and *myh10* null zebrafish mutant lines using CRISPR genome editing. Through examination of gross morphology and survival in our mutants we discovered that *myh9b* is the critical NMII encoding gene required for normal zebrafish development. Additionally, we determined there are genetic interactions between the *myh9* and *myh10* genes. We found that *myh9b* is required for expression of the *myh9a* gene and that the *myh9b* and *myh10* genes are both required for expression of each other. Genetic interactions were further explored through the examination of double mutant lines where more severe phenotypes were found to develop at earlier time points than were observed in single mutant counterparts. Double mutant phenotype investigation suggested roles for tissue specific genetic redundancy and haploinsufficiency in some genotypes. This work expands on the current understanding of the NMII encoding *myh* genes in development, establishes genetic interactions occurring between them, and provides novel loss-of-function mutant models for the study of the NMIIA and NMIIB encoding *myh* genes.

## Material and methods

### Zebrafish maintenance and husbandry

Standard procedures were used for zebrafish maintenance and husbandry (Kimmel *et al*. 1995; Westerfield 2000). Zebrafish lines used included: wild-type (WT) AB strain; *myh9a^sa20632^* mutant line that was obtained through the Sanger Zebrafish Mutation Project (Busch-Nentwich et al. 2012); *myh9b^mke414^* and *myh10^mke508^* lines that were generated as a part of this study (see details below). This work was conducted in compliance with all regulations set forth by the University of Wisconsin–Milwaukee Institutional Animal Care and Use Committee (IACUC). The University of Wisconsin–Milwaukee is accredited by the American Association for Accreditation of Laboratory Animal Care (AAALAC).

For line maintenance, *myh9a^sa20632^* mutants were genotyped through sequencing. *myh9b^mke414^* and *myh10^mke508^* mutants were genotyped by PCR product agarose gel analysis. Primers used for *myh* mutant genotyping can be found in Supplementary Table S1. All oligonucleotides used for these studies and in Supplementary materials were purchased from Eurofins Genomics unless otherwise indicated.

### Gene and protein homology studies

Sequence information was obtained for zebrafish and human from the Ensembl genome browser (GRCz11 and GRCh38.p14) (Howe *et al*. 2021; Cunningham *et al*. 2022). Zebrafish transcript IDs used for these studies can be found in Supplementary Table S2. All homology studies were completed using sequence alignments in Clustal Omega (Sievers *et al*. 2020). The zebrafish *myh9b* (202) ENSDART00000137105.3 transcript contains an incomplete 3′ annotation. Therefore, homology studies for *myh9b* (NMIIA) were conducted by combining our 3′RACE data (Supplementary Fig. S1) with the available *myh9b* sequence in Ensembl.

### Zebrafish mutant generation

*myh9b^mke414^* and the *myh10^mke508^* mutant lines were generated using CRISPR/Cas9 mutagenesis according to (Talbot and Amacher 2014) and (Gagnon *et al*. 2014). Briefly, Ensembl was used to obtain sequence information for the *myh9b* and *myh10* zebrafish genes (transcript IDs are in Supplementary Table S2) and gRNAs were designed using CHOPCHOP (Montague *et al*. 2014; Labun *et al*. 2016, 2019). Specific CRISPR target sequence information used for genome editing can be found in Supplementary Table S3. For the *myh10* CRISPR mutant generation we included the stop codon cassette as published by Gagnon *et al*. 2014 with *myh10* region specific homology arms: 5′-tcgagatgtacaggggcaagGTCATGGCGTTTAAACCTTAATTAAGCTGTTGTAGaagaggcacgagatgccgcc-3′.

The gene specific and constant oligonucleotides were annealed as described in Gagnon *et al*. 2014 and in vitro transcription reactions were completed using the T7 Megashortscript kit (CAT #AM1354, Thermo Fisher Scientific). Cas9 was obtained from Addgene (plasmid #47322) (Gagnon *et al*. 2014). mRNA was in vitro transcribed from the Cas9 plasmid using the SP6 message machine kit (CAT #AM1340, Thermo Fisher Scientific). CRISPR injections were conducted by injection of the following mixes directly into the single-cell of WT embryos. For generation of the *myh9b^mke414^* mutant line, injection of 1 nl of the following mix per embryo: 100–400 ng/μl of Cas9 mRNA, 200–400 ng/μl of sgRNA in nuclease-free H_2_O. For generation of the *myh10^mke508^* mutant line, injection of 1 nl of the following mix per embryo: 100–400 ng/μl Cas9 mRNA, 200–400 ng/μl sgRNA, with 3 mM of the stop codon oligonucleotide. Following injections, embryos were allowed to develop until 24 hours post fertilization (hpf) at which time 10 embryos from each clutch were collected for screening using qPCR with high resolution melt analysis (HRMA) according to (Talbot and Amacher 2014). HRMA primers were designed using Primer3 (Koressaar and Remm 2007; Untergasser *et al*. 2012; Kõressaar *et al*. 2018). HRMA primer sequences can be found in Supplementary Table S4. Next, germline transmission was screened in adult progeny, and F0 germline transmission founders were outcrossed to WT adults for two additional generations prior to analysis of progeny.

### Mutant survival analysis

The WT population was generated via WT adult incrosses. *myh9a* and *myh10* populations were generated via *myh9a^−/−^* and *myh10^−/−^* adult incrosses, respectively. The *myh9b* population was generated via *myh9b^+/−^* parent incrosses and the *myh9b^−/−^* mutant population was calculated based on the collection and genotyping of fish that died during the study and those that survived through 40 days post fertilization (dpf). Offspring were collected from at least three separate parent crosses for each genotype examined and pooled to generate the four survival study populations. Zebrafish were maintained in an incubator (28°C) through 5 dpf and then transferred into fish tanks and were counted daily through 40 dpf. All fish in this study were fed at the same time and same amount each day.

### Bright-field imaging and mutant phenotype analysis

Bright-field images were taken using an Olympus SZX12 with an Olympus U-CMAD3 camera mount (Falat *et al*. 2022). WT offspring were generated via WT parent incrosses. *myh9a^−/−^* and *myh10^−/−^* mutant populations were generated via *myh9a^−/−^* or *myh10^−/−^* parent incrosses, respectively. *myh9b^−/−^* mutants were generated via *myh9b^+/−^* parent incrosses and offspring were genotyped after image acquisition. Phenotype reversal data for the *myh9b* mutants was generated from *myh9b^+/−^* parent incrosses and the pericardial edema phenotype that developed was counted between 48 hpf through 6 dpf following the same groups of fish over time.

### *myh9b* mutant qRT-PCR at 96 hpf

Larvae were generated either via homozygous mutant parent incrosses or through heterozygous parent incrosses. Populations produced through heterozygous parent incrosses were tail-clipped, genotyped, and then pooled based on genotype for RNA isolation. Each biological replicate contained 6 larvae at 96 hpf and RNA was treated with DNAseI prior to cleanup using the RNA clean and concentrator-5 kit Zymoclean (CAT #R1013, Zymo). cDNA was generated for each replicate using 50 ng of total RNA (CAT #18080-051, Thermo Fisher). All primers were designed using Primer3 and tested for specificity in targeting the desired gene using the blast function in Ensembl and by examining predicted product size. Optimal annealing temperatures for each qRT-PCR primer pair was determined for each gene analyzed, and the *eef1al1* gene was used as a control gene and for normalization. qRT-PCR was performed with SYBR green (CAT #1725112, BioRad) on a BioRad CFX96 Real-Time System. Relative quantification of gene expression was done using the 2^−ΔΔCT^ method (Livak and Schmittgen 2001). qRT-PCR primer information can be found in Supplementary Table S5.

### Western Blot analysis at 96 hpf

Each biological replicate contained 50 larvae of each genotype at 96 hpf. Protein was denatured and 30 μg of protein isolated from whole larvae were loaded onto 4–20% BioRad mini protean TGX gels (CAT #4561094, BioRad). Protein was transferred to PVDF membrane, and then the membrane was blocked in 5% skim milk/1× TBST on a shaker at room temperature for 1 h. Blot was incubated in primary antibodies overnight on a rocker at 4°C. NMIIA primary antibody was diluted in 5% milk/1× TBST. NMIIB and alpha-tubulin primary antibodies were diluted in porcine skin gelatin powder/1× TBST. Secondary antibodies were diluted in 5% milk/1× TBST and blot was incubated in secondary antibody at room temperature on a shaker for 2 h. Antibodies used include: NMIIA (ab55456, Abcam) at 1:2,000, NMIIB (sc-376942, Santa Cruz Biotechnology) at 1:500, and alpha-tubulin (T6199, Sigma) at 1:2,000 dilutions. Secondary antibodies were anti-mouse IgG horseradish peroxidase (Cell Signaling Technology 7076S) at 1:2,000 dilutions (Visetsouk *et al*. 2018). Signal was analyzed in Adobe Photoshop. Quantification of three independent biological replicates and Western experiments are represented. NMIIA and NMIIB signals were normalized to alpha-tubulin. Each sample was standardized to WT protein level.

### Whole-mount in situ hybridization

In situ hybridization was conducted according to standard procedures in WT zebrafish at the indicated time points. RNA probes used for *myh9a*, *myh9b*, and *myh10* were previously described in (Gutzman *et al*. 2015). Sense and antisense probes were used for each experiment. Total numbers of fish analyzed at each time point can be found in Supplementary Table S6.

### Statistics

Statistics were calculated in Microsoft Excel (16.83). Survival curves were generated using the Kaplan–Meier method and log-rank tests were used to determine statistical differences in survival. For *myh9a*, *myh9b*, and *myh10* gene expression analyses and NMIIA and NMIIB protein expression analyses, one tailed *t*-tests were conducted to test for samples with equal variance. The false discovery rate was controlled for comparisons using the Benjamini–Hochberg method with a false discovery control level *α* = 0.05 and corrected *P*-values are reported within the body of the text. For mutant phenotype analysis, we hypothesized that homozygous mutants produced from each cross would develop phenotypes. The *χ*^2^ tests were done to determine whether the phenotype development observed was Mendelian based on parental genotypes.

## Results

### NMII protein structure, gene and protein homology, and expression during larval zebrafish development

**Fig. 1.**
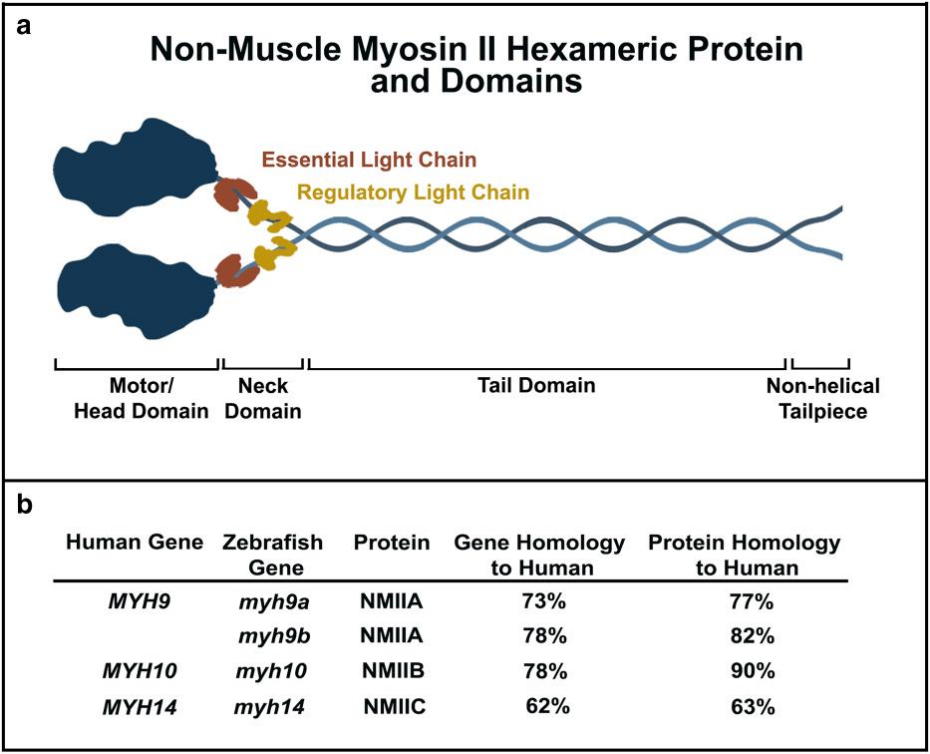
General NMII protein structure and homology. a) Schematic of the hexameric structure of an NMII protein. b) Zebrafish to human gene and protein homology. Zebrafish contain two *myh9* genes, *myh9a*, and *myh9b*, compared to human *MYH9*.

NMII proteins function as hexameric structures, consisting of two heavy chains (encoded by the *MYH* genes), two essential light chains, and two regulatory light chains (Dulyaninova and Bresnick 2013; Fig. 1a). The regulatory light chains are phosphorylated through upstream signaling pathways to regulate NMII activity (Garrido-Casado *et al*. 2021). The NMII proteins contain an *N*-terminal motor domain, a neck domain for light chain protein binding, a coiled-coil tail domain, and a nonhelical tailpiece (Fig. 1a) (Billington *et al*. 2013). The motor domains are highly conserved among the three NMII isoforms and contain actin binding sites and ATPase domains (Cope *et al*. 1996; Sellers and Heissler 2019). The distant C-terminal domain contains the greatest sequence diversity and contributes to isoform specific roles, such as the determination of the cellular distribution of the NMIIA and NMIIB proteins (Sandquist and Means 2008; Ronen and Ravid 2009).

Sequence homology studies were completed to determine the feasibility of using zebrafish as a model system to explore the role of the NMII encoding *MYH* genes in development (Fig. 1b). Humans express three NMII coding *MYH* genes *MYH9*, *MYH10*, and *MYH14*, which encode the NM IIA, IIB, and IIC protein isoforms, respectively. However, due to the teleost genome duplication, zebrafish express two *myh9* genes, *myh9a*, and *myh9b*, both of which encode NMIIA isoforms. When comparing gene sequences, it was discovered that the current *myh9b* gene annotation in Ensembl is incomplete (ENSDART00000137105.3). Therefore, 3′RACE experiments were completed and combined with the available *myh9b* sequence in Ensembl to determine the 3′-coding sequence for the *myh9b* gene. This sequence was used for determination of gene and protein homology for *myh9b* (NMIIA) (Fig. 1b and Supplementary Fig. S1). We found high gene and protein homology comparing zebrafish to human for *myh9a* (NMIIA), *myh9b* (NMIIA), and *myh10* (NMIIB) (>73%), but not *myh14* (NMIIC) (<63%). Due to the lower homology of the zebrafish *myh14* gene and NMIIC protein to human, low levels of *myh14* mRNA expression in early zebrafish development (White *et al*. 2017), and lack of phenotypes observed in previously developed null NMIIC mouse models (Ma *et al*. 2010), the role of *myh14* in development was not examined in this study.

**Fig. 2.**
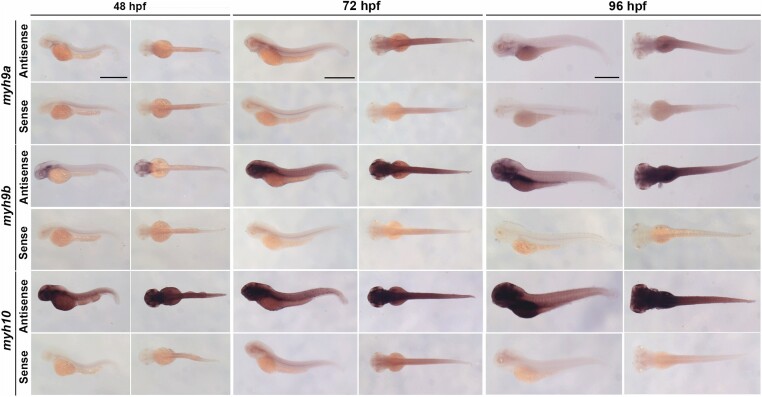
*Myh* expression in WT larvae by whole-mount in situ hybridization for *myh9a*, *myh9b*, and *myh10* at 48, 72, and 96 hours post fertilization. Antisense probe expression pattern and sense controls shown for each time point. Scale bars = 1 mm and apply to all images for the same time point.

Before examining gene function and potential interaction, we expanded on our previous work to identify the localization of *myh9a*, *myh9b*, *and myh10* gene expression in developing zebrafish embryos and larvae beyond 24 hours post fertilization (hpf) (Gutzman *et al*. 2015). Whole-mount in situ hybridization experiments were conducted in WT zebrafish at 48, 72, and 96 hpf (Fig. 2). *myh9a* showed low levels of expression at all time points analyzed, which is consistent with our earlier report (Gutzman *et al*. 2015). *myh9b* was found to be expressed at all time points analyzed. At 48 hpf, *myh9b* was found mostly in the head region, but at 72 and 96 hpf it was found to be broadly expressed throughout both the head and trunk. *myh10* was also highly expressed at all time points analyzed in the head and trunk. Nonspecific staining was found in the notochord for all three genes based on sense control experiments, and in the head and trunk region in *myh10* control experiments. These studies indicate that *myh9b* and *myh10* remain highly and broadly expressed through 5 dpf, while *myh9a* expression remains low, consistent with expression patterns found during early embryonic development (Gutzman *et al*. 2015). This expression localization led us to hypothesize that *myh9b* and *myh10* would be critical genes throughout embryonic and larval development.

### *myh9b* is the critical NMII encoding *myh* gene required for normal zebrafish development

Zebrafish *myh9a*, *myh9b*, and *myh10* null mutants were either obtained or generated to investigate the role of each gene during zebrafish development. Details for each mutant line are outlined in Table 1 and mutation schematics are shown in Supplementary Fig. S2. The *myh9a* mutant (*myh9a^sa20632^*) was obtained from the Sanger Institute Zebrafish Mutation Project and contains an A>T base pair (bp) change resulting in an early stop codon in exon 10, located in the head domain of the NMIIA protein (Busch-Nentwich et al. 2012). The *myh9b* (*myh9b^mke414^*) mutant was generated for this study using CRISPR/Cas9 genome editing and contains a 3 bp insertion and a 34 bp deletion in exon 11, resulting in a frameshift mutation causing an early stop in the head domain of the NMIIA protein. The *myh10* (*myh10^mke508^*) mutant was also generated by CRISPR/Cas9 genome editing for this study. It contains the insertion of a stop codon cassette as designed by (Gagnon *et al*. 2014), and results in a 51 bp insertion and 4 bp deletion, creating an early stop in the head domain of the NMIIB protein.

**Table 1.** Zebrafish *myh9a*, *myh9b*, and *myh10* mutant lines used in this study.

Mutant allele	Mutation type	Location	Result
***myh9a^sa20632^***	ENU Nonsense point mutation A > T	Exon 10 (Head domain)	Early stop at A.A 363
***myh9b^mke414^***	CRISPR generated; 3 bp insertion, 34 bp deletion	Exon 11 (Head domain)	Early stop at A.A 431 due to frameshift mutation
***myh10^mke508^***	CRISPR generated; 51 bp insertion with STOP cassette knock-in, 4 bp deletion	Exon 3 (Head domain)	Early stop at A.A 148 due to insertion of stop cassette

*myh9a^sa20632^* mutant line was obtained from the Sanger Zebrafish Mutation Project (Busch-Nentwich et al. 2012). *myh9b^mke414^* and *myh10^mke508^* mutant lines were generated using CRISPR/Cas9 mutagenesis, see Materials and Methods.

**Fig. 3.**
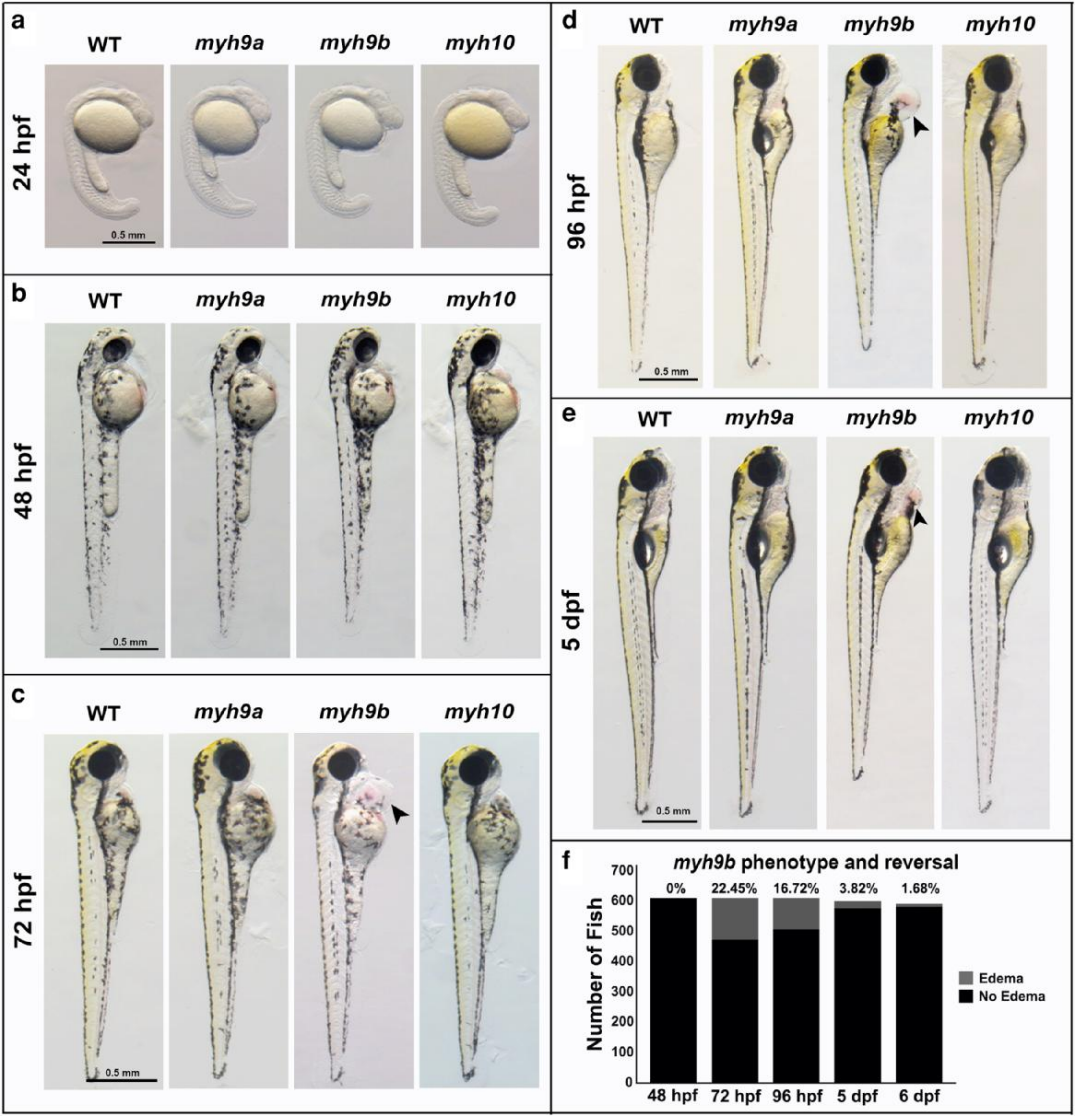
*myh^−/−^* mutant phenotypes between 24 hpf and 5 dpf. a–e) Representative bright-field images of *myh9a^−/−^*, *myh9b^−/−^*, and *myh10^−/−^* homozygous mutants compared to WT at 24 hpf, 48 hpf, 72 hpf, 96 hpf, and 5 dpf. Arrowheads indicate location of pericardial edema in *myh9b^−/−^* mutants. f) Quantification of *myh9b* pericardial edema phenotype development and reversal from 48 hpf to 6 dpf in offspring generated from an *myh9b^+/−^* parent incross. The *χ*^2^ analyses determined that at 72 hpf the edema phenotype development is Mendelian, and at 96 hpf and beyond it is no longer Mendelian. Scale bars for all images = 0.5 mm. *n* = 610 for phenotype development and reversal quantification in f.

To explore the role of the NMIIA and NMIIB proteins in development, *myh9a^−/−^*, *myh9b^−/−^*, and *myh10^−/−^* mutant zebrafish were observed for gross morphological defects between 24 hpf and 5 dpf (Fig. 3a-e). *myh9a* and *myh10* homozygous mutant embryos and larvae appeared phenotypically indistinguishable from WT at all time points observed (Fig. 3). In addition, *myh9a^−/−^* and *myh10^−/−^* adults are viable, fertile, and appeared indistinguishable from WT. *myh9b^−/−^* mutant embryos appeared normal through 48 hpf (Fig. 3a and b). However, between 48 hpf and 72 hpf *myh9b^−/−^* mutants developed pericardial edema, which was still visible in many fish at 96 hpf (Fig. 3c and d and Supplementary Fig. S3). We discovered this phenotype reverses and is largely resolved in the majority of mutants by 5 dpf (Fig. 3e).

We quantified the onset and reversal of pericardial edema by examining offspring generated from *myh9b^+/−^* parent incrosses. We found that by 72 hpf, 22.45% of the offspring developed this edema (Fig. 3c and f). While following the same fish over time, at 96 hpf, only 16.72% of offspring had the edema (Fig. 3d and f). And at 96 hpf, some offspring had reversal of this edema, while a small number (6 fish) had late onset development of edema. By 5 dpf, a small percentage of fish still had edema (Fig. 3f), with varying degrees of severity (Fig. 3e, fish with mild lingering edema pictured and Supplementary Fig. S3). Together, 144 out of 610 fish observed developed the pericardial edema phenotype, for a total of 23.6% of the population. Overall, 133 of the 144 fish (92.3%) that developed pericardial edema experienced a reversal of the phenotype by 6 dpf. Fish incapable of phenotypic reversal were typically those that developed more severe edema than pictured (Fig. 3c and d). The *χ*^2^ analysis confirmed that at 72 hpf, the phenotype is Mendelian; however, at 96 hpf and beyond, Mendelian ratios were no longer present. This phenotypic reversal of the pericardial edema phenotype, with no other obvious gross morphological phenotypes present, led us to ask whether *myh9b^−/−^* mutants were viable. It also led to questions regarding the presence of genetic compensation or redundancy by *myh9a* or *myh10* in *myh9b^−/−^* mutants. Together, these three zebrafish lines represent the first in vivo vertebrate models that allow for the study of *myh9* and *myh10* loss-of-function mutations in the motor domain of their respective NMIIA and NMIIB proteins during embryonic and larval development and provide the tools needed to test for genetic interactions.

### *myh9b* homozygous mutants have a partially penetrant lethal phenotype

**Fig. 4.**
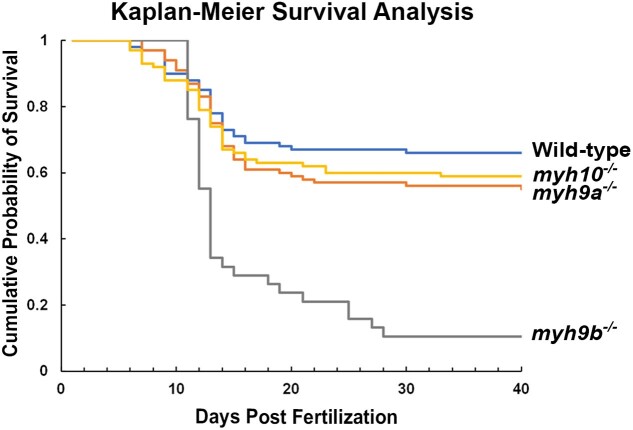
Kaplan–Meier survival curve for the *myh^−/−^* mutants through 40 dpf. Only *myh9b^−/−^* mutants demonstrated decreased survival rates compared to WT, *myh9a^−/−^*, and *myh10^−/−^* mutant populations (log-rank tests *P* < 0.001). WT, *n* = 100. *myh9a^−/−^*, *n* = 100. *myh10^−/−^*, *n* = 100. *myh9b^−/−^*, *n* = 38.

We determined that *myh9a^−/−^* and *myh10^−/−^* mutants grow into viable and fertile adults, but we were unable to successfully rear *myh9b^−/−^* mutants to adulthood. Therefore, we compared survival rates in each of the homozygous mutant populations to WT (Fig. 4). WT populations were generated through WT parent incrosses. *myh9a^−/−^* and *myh10^−/−^* populations were generated through homozygous mutant parent incrosses of the respective genotypes. *myh9b^−/−^* populations were generated through *myh9b^+/−^* incrosses, and the *myh9b^−/−^* population was identified based on collecting and genotyping fish that died throughout the study and those that survived to the end of the study, past 40 dpf. We found no survival differences between WT, *myh9a^−/−^*, and *myh10^−/−^* mutant populations through 40 dpf (Fig. 4). However, Kaplan–Meier Log Rank tests demonstrated that *myh9b^−/−^* mutants have significantly decreased survival rates compared to all three groups *P* < 0.001 (*myh9b^−/−^* compared to WT, *myh9a^−/−^*, and *myh10^−/−^*) (Fig. 4). Notably, the *myh9b^−/−^* survival data at 40 dpf is not reflective of overall survival to adulthood. While we did not quantify survival after 40 dpf, in our attempts to selectively raise *myh9b^−/−^* mutant zebrafish based on pericardial edema phenotype development at 72 hpf (Fig. 3c), we found only a very small quantity of *myh9b^−/−^* mutants (<10 fish) survived to adulthood. Of these surviving adults, the mutant fish appeared to be smaller in size and had a limited life span, as no adult fish lived longer than 1.5 years, and the majority died at or before 6 months of age. These *myh9b^−/−^* escapers were able to successfully produce offspring when mated with WT zebrafish but attempts at *myh9b^−/−^* incrosses were unsuccessful. These observations suggest that *myh9b*^−/−^ mutant adults were fertile, but likely contain underlying conditions that may have impacted their survival. Finally, we did not identify any survival defects in our *myh9b^+/−^* mutants compared to WT, which is supported by our ability to raise and identify this genotype. The *myh9b^+/−^* mutants also exhibited high fecundity.

### *myh9b* is required for expression of *myh9a* and *myh10* during larval development

**Fig. 5.**
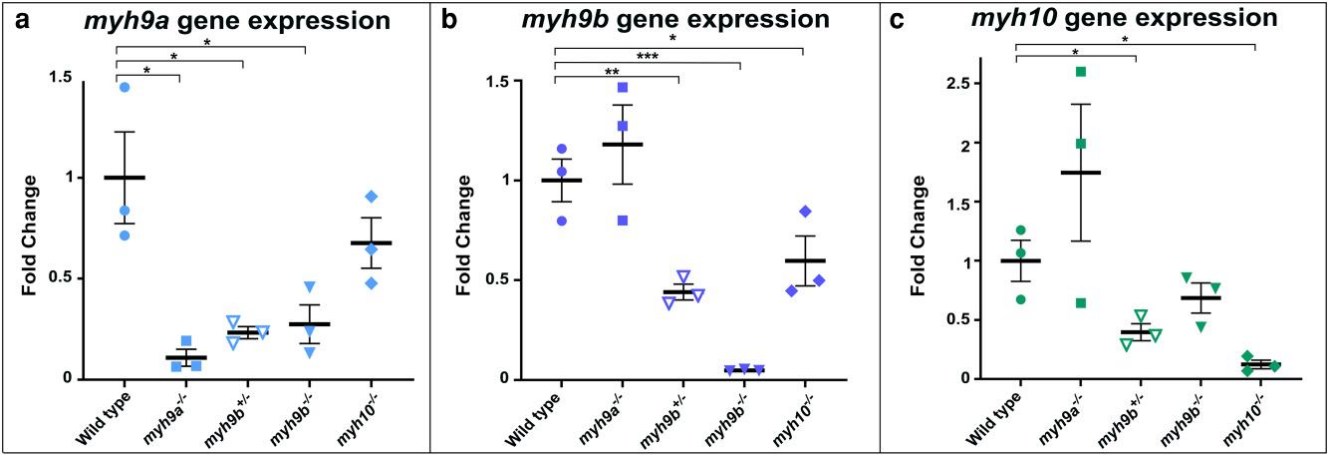
*myh9a*, *myh9b*, and *myh10* gene expression in zebrafish *myh* mutants at 96 hpf. Whole larvae qRT-PCR. a) Quantification of *myh9a* gene expression for each of the *myh* mutants. b) Quantification of *myh9b* gene expression for each of the *myh* mutants. c) Quantification of *myh10* gene expression for each of the *myh* mutants. The *eef1al1* gene was used for data normalization to determine fold change. +/− SEM is shown for all graphs. *N* = 3 independent experiments for each gene analyzed. *P < 0.05, **P < 0.01, and ***P < 0.005.

We found that *myh9a^−/−^* and *myh10^−/−^* mutants did not develop gross morphological phenotypes at the time points observed (Fig. 3), did not have survival differences (Fig. 4), and were viable and fertile adults. In contrast, we discovered that the *myh9b^−/−^* mutants developed pericardial edema (Fig. 3) and have a partially penetrant lethal phenotype (Fig. 4). These results further supported our hypothesis that genetic compensation or genetic redundancy may be occurring in these mutants. To investigate this, whole larvae qRT-PCR was performed at 96 hpf to examine expression of *myh9a*, *myh9b*, and *myh10* in the mutant genotypes shown (Fig. 5). The 96 hpf time point was selected for analysis as it occurs within the window of *myh9b* mutant pericardial edema phenotype reversal. Therefore, we hypothesized if genetic compensation was occurring, it would be possible to capture it at this time point. qRT-PCR primers were designed and confirmed for specificity in targeting each gene of interest using Primer3 for design and the zebrafish genome Ensembl (GRCz11) blast function to confirm target specificity. Optimal annealing temperatures were determined for each primer pair and gel electrophoresis further confirmed amplicon size was as predicted. Fold change analysis was done by normalizing each gene of interest to the control *eef1al1* gene. *eef1al1* gene expression levels were plotted for each genotype at 96 hpf (Supplementary Fig. S4).

The *myh9a^−/−^*, *myh9b^−/−^*, and *myh10^−/−^* mutants had less *myh9a*, *myh9b*, and *myh10* expression, respectively, compared to WT, likely due to nonsense mediated decay and confirming they are null mutants for their respective genes (Fig. 5). Interestingly, in the *myh9b^+/−^* and *myh9b^−/−^* mutant populations there was a significant decrease in *myh9a* expression (*P* = 0.03, and *P* = 0.04, respectively) (Fig. 5a), which suggested that the *myh9b* gene is required for the expression of *myh9a*. There was no significant change in *myh9a* expression in *myh10^−/−^* mutants (Fig. 5a). Examination of *myh9b* expression in each of the *myh* mutant lines revealed decreased *myh9b* expression in the *myh10^−/−^* mutants compared to WT (*P* = 0.04) (Fig. 5b), which suggested that the *myh10* gene is required for the expression of *myh9b*. Finally, we examined *myh10* gene expression in the mutant groups. Interestingly, while the *myh9b^−/−^* mutant line appeared to have less *myh10* gene expression, only the *myh9b^+/−^* mutant line showed significant decreases (*P* = 0.04) (Fig. 5c). The decreased expression of the *myh10* gene in the *myh9b* mutant line suggested that *myh9b* is required for the expression of *myh10*. Together, these results suggested genetic interactions where *myh9b* is required for the expression of both *myh9a* and *myh10*, while *myh10* is required for the expression of *myh9b*. However, these effects may also be due to an indirect mechanism. For example, the development of the organism could be generally compromised; whereby an indirect reduction in transcript levels could occur.

### Increased NMIIA protein level in *myh10^−/−^* mutants may indicate compensation through a potential change in protein stability

**Fig. 6.**
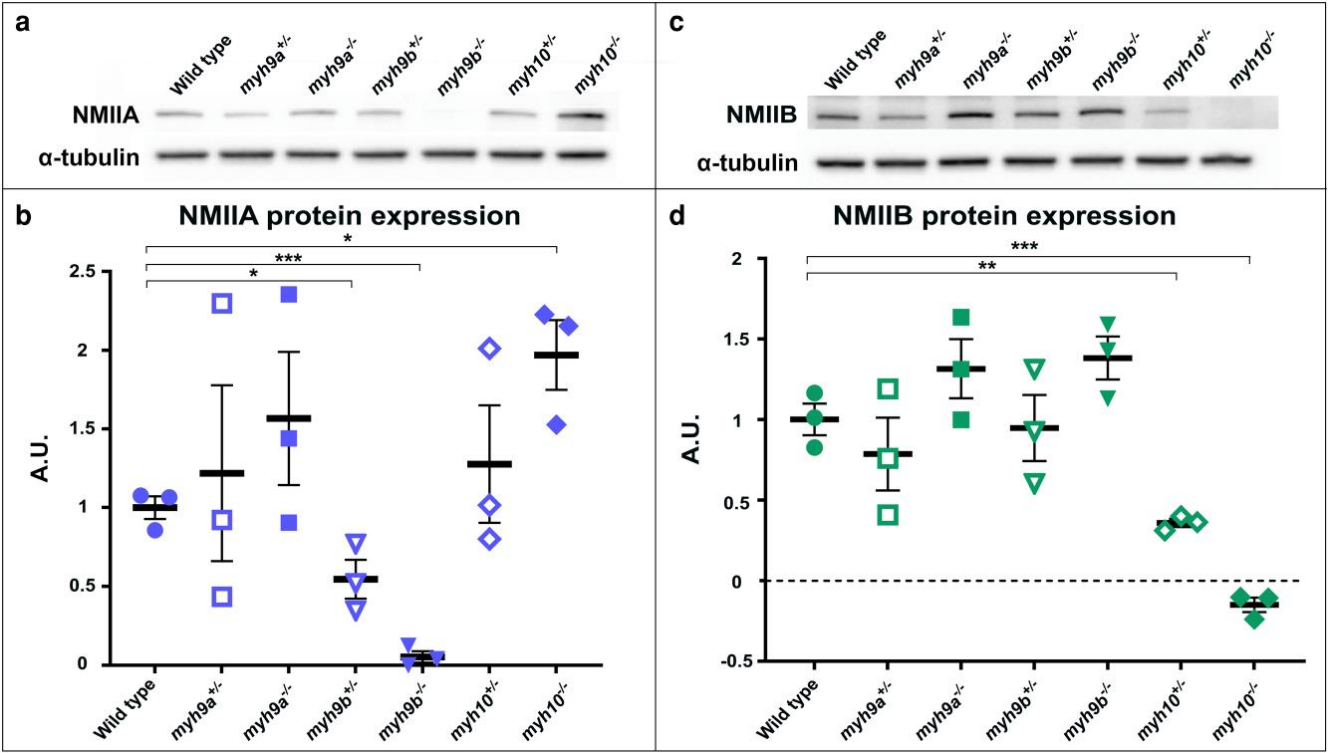
NMIIA and NMIIB protein levels in the zebrafish *myh* mutants. a) Representative western blot of whole larvae extracts at 96 hpf for NMIIA protein with alpha-tubulin control for each of the *myh* mutants. b) Quantification of NMIIA protein in the *myh* mutants. c) Representative western blot of whole larvae extracts at 96 hpf for NMIIB protein with alpha-tubulin control for each of the *myh* mutants. d) Quantification of NMIIB protein in the *myh* mutants. +/− SEM bars shown for all graphs. *N* = 3 independent experiments for each protein analyzed. *P < 0.05, **P < 0.01, and ***P < 0.005.

Next, we examined NMIIA and NMIIB protein levels in the *myh* mutant lines (Fig. 6). We found loss of NMIIA protein in *myh9b* mutant lines (*myh9b^+/−^*, *P* = 0.04 and *myh9b^−/−^*, *P* = 0.003), consistent with their genotype; however, no significant change in NMIIA protein levels were detected in the *myh9a* mutants (Fig. 6a and b). We hypothesize that the nonobservable change in NMIIA protein in the *myh9a* mutants is due to antibody recognition. With limited availability of commercial antibodies targeting zebrafish proteins, and the high homology between the zebrafish NMIIA proteins produced from both the *myh9a* and *myh9b* genes (82.06%), we used an NMIIA antibody targeting human NMIIA (Abcam, ab55456). This antibody was made against NMIIA amino acids 1871–1960, within the tail domain of the human protein. We predict that only the *myh9b* encoded NMIIA protein product was detected with this reagent. Alignments comparing the NMIIA antibody to the NMIIA proteins produced from both the *myh9a* and *myh9b* genes revealed that the NMIIA sequence from *myh9a* is 63.22% identical while the NMIIA sequence from *myh9b* is 71.26%, establishing a possibility for differential antibody binding efficiency between the NMIIA protein products. This provided an explanation for why there are no detectable NMIIA protein changes in the *myh9a* mutant lines. Although, we were unable to assay expression of the NMIIA protein produced by the *myh9a* gene, our qRT-PCR data demonstrated that *myh9a* mutants lacked *myh9a* gene expression (Fig. 5a), confirming they are a feasible mutant for studies of *myh9a* gene.

Before examining NMIIA levels, we hypothesized that NMIIA would decrease in *myh10^−/−^* mutants, following the gene expression pattern observed in Fig. 5. However, we identified increased NMIIA levels in the *myh10^−/−^* mutant line compared to WT (*P* = 0.03) (Fig. 6a and b). These data suggested compensation by NMIIA for loss of *myh10*. This poses the question as to how there could be an increased level of the NMIIA protein, but decreased *myh9b* gene expression, in the *myh10^−/−^* mutants (Kumar *et al*. 2018). There does appear to be a slight, although not significant, increase in NMIIA protein levels in *myh9a^−/−^* mutants, which may also indicate potential compensation by *myh9b* with loss of *myh9a*.

Examination of NMIIB protein levels in each of the *myh* mutant groups demonstrated decreased NMIIB protein expression in both the *myh10^+/−^* and *myh10^−/−^* mutant lines compared to WT (*P* = 0.005 and *P* = 0.002, respectively) as expected (Fig. 6c and d). There were no significant differences in NMIIB protein expression in the *myh9a* or *myh9b* mutant lines. However, as with NMIIA, there were trends toward increased NMIIB protein levels in both *myh9a^−/−^* and *myh9b^−/−^* mutants (Fig. 6c and d). Together, these data demonstrated changes in protein levels without corresponding changes in gene expression in *myh10* mutants, as well as trends in *myh9b* mutants. These findings highlight the potential for a compensatory mechanism of indirect modulation to increase protein stability with loss of *myh* gene expression. However, the identifying the mechanisms underlying this protein compensation were outside the scope of this study.

### Double mutant phenotypes further indicate genetic interactions between *myh9a*, *myh9b*, and *myh10*

To further explore potential genetic interactions we generated double mutants, by selectively crossing our *myh9a*, *myh9b*, and *myh10* single mutant lines to produce *myh9a;myh9b*, *myh9a;myh10*, and *myh9b;myh10* double mutant lines. There were no double homozygous mutant combinations that were viable to adulthood. The only double mutant genotype adult populations that were viable and used to produce offspring for the following studies included: *myh9a^−/−^*; *myh9b^+/−^*, *myh9a*^+/−^; *myh10*^+/−^, and *myh9b*^+/−^; *myh10*^−/−^.

**Fig. 7.**
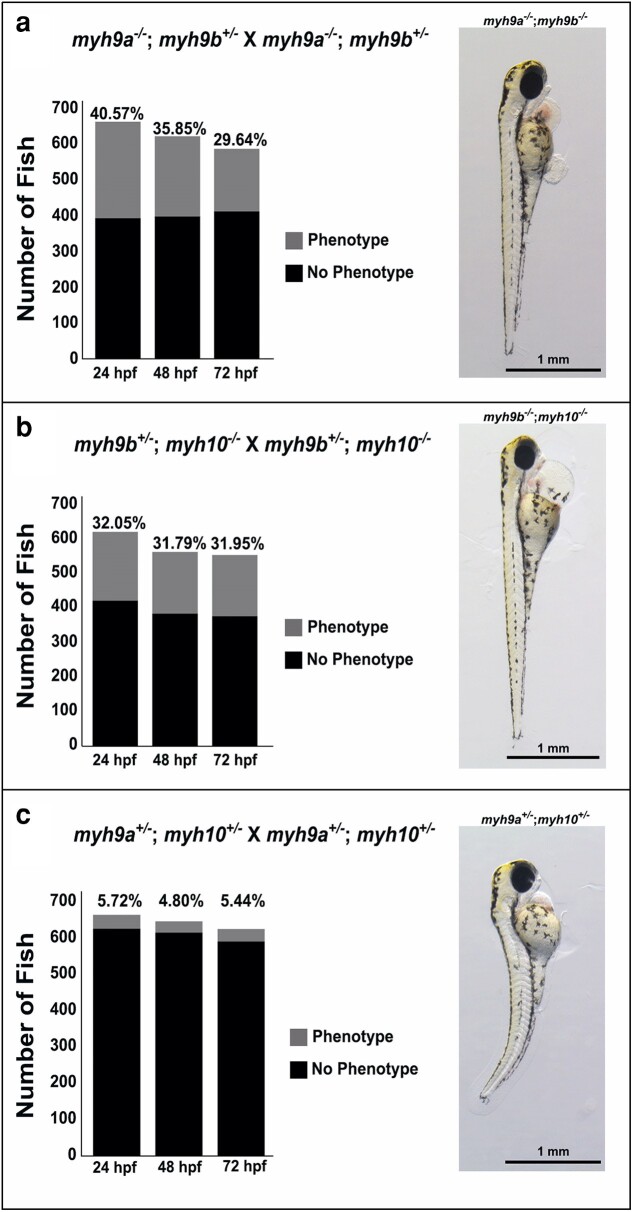
Double mutant phenotype quantification between 24 and 72 hpf and representative images at 72 hpf. a) Phenotype quantification for *myh9a^−/−^*; *myh9b^+/−^*double mutant parent incrosses and representative image of a 72 hpf double homozygous mutant. The *χ*^2^ calculations determine at 24, 48, and 72 hpf *myh9a;myh9b* double mutant phenotype development is non-Mendelian. b) Phenotype quantification for *myh9b^+/−^*; *myh10^−/−^* double mutant parent incrosses and representative image of a 72 hpf double homozygous mutant. The *χ*^2^ calculations determine at 24, 48, and 72 hpf *myh9b;myh10* double mutant phenotype development is non-Mendelian. c) Phenotype quantification for *myh9a^+/−^*; *myh10^+/−^* double mutant parent incrosses and representative image of a 72 hpf double heterozygous mutant. The decrease in numbers throughout each evaluation period in a–c) was due to death among the population of fish studied.

Crossing *myh9a^−/−^*; *myh9b^+/−^* X *myh9a^−/−^*; *myh9b^+/−^* (Fig. 7a and Table 2) revealed double mutant phenotypes by 24 hpf. This is in contrast to the lack of observable phenotypes in *myh9a*^−/−^ single mutants and the later pericardial edema in the *myh9b^−/−^* single mutants. Commonly observed double mutant phenotypes included: shortened yolk extensions, heart edema, yolk edema, fluid filled cysts, and skin blistering. Representative *myh9a^−/−^*; *myh9b^−/−^* larva is shown at 72 hpf with heart edema and skin blistering around the yolk (Fig. 7a) (additional phenotype images are shown in Supplementary Fig. S5). From this cross, we expected 25% of the progeny to be double homozygous mutants and present with phenotypes. The *χ*^2^ analyses were completed, and non-Mendelian ratios were found at all time points analyzed (Fig. 7a). Additionally, progeny presenting with a phenotype were genotyped and it was confirmed that more than just the *myh9a^−/−^; myh9b^−/−^* mutant population developed phenotypes. These data indicate that in the *myh9a^−/−^* background, embryos are haploinsufficient for *myh9b^+/−^*.

**Table 2.** Primary observed phenotypes from double mutant crosses.

Double mutant cross	Primary observed phenotypes
*myh9a^−/−^*; *myh9b^+/−^* X *myh9a^−/−^*; *myh9b^+/−^*	Heart edema Yolk edema Fluid filled cysts Shortened yolk extensions Skin blistering
*myh9b^+/−^*; *myh10^−/−^* X *myh9b^+/−^*; *myh10^−/−^*	Heart edema Yolk edema Fluid filled cysts Developmental arrest Necrotic tissue Curved or curled body axes Male infertility
*myh9a^+/−^*; *myh10^+/−^* X *myh9a^+/−^*; *myh10^+/−^*	Heart edema Yolk edema Small size Curved tails

Double mutant analyses also indicated that progeny from *myh9b^+/−^*; *myh10^−/−^* X *myh9b^+/−^*; *myh10^−/−^* crosses developed phenotypes by 24 hpf, again much earlier than the single *myh9b^−/−^* mutants (Fig. 7b and Table 2). Common phenotypes observed included: developmental arrest, regions of necrotic tissue, heart edema, yolk edema, fluid filled cysts, and curved or curled body axes. Representative *myh9b^−/−^*; *myh10^−/−^* larva is shown at 72 hpf and contains severe heart edema, a common phenotype that typically worsened and progressed to full body edema in these fish after 72 hpf (Fig. 7b and Table 2). From this cross, we again would expect 25% of the progeny to be double homozygous mutant and present with phenotypes. The *χ*^2^ analyses were completed, and non-Mendelian ratios were found at all time points analyzed (Fig. 7b). Progeny presenting with a phenotype were also genotyped and it was confirmed that more than just *myh9b^−/−^*; *myh10^−/−^* mutants developed phenotypes. These data indicate haploinsufficiency was also occurring in *myh9b;myh10* mutant populations.

Through these studies, we discovered that the *myh9b^+/−^*; *myh10^−/−^* double mutant adult males had fertilization defects. We crossed *myh9b^+/−^*; *myh10^−/−^* double mutant males, from two different generations, to WT females, and found that out of 851 eggs, 488 were fertilized while 403 were unfertilized. We did not observe any fertilization defects when we crossed *myh9b^+/−^*; *myh10^−/−^* double mutant females to WT males. Therefore, we predict that this male fertility defect is due to abnormal spermatogenesis in the *myh9b^+/−^; myh10^−/−^* adult males. This is supported by previously published work in mice establishing the requirement of NMIIB in male, but not female fertility, as it is required for cytokinesis during meiotic cell division (Yang *et al*. 2012). We did not observe any obvious fertility defects in *myh9b^−/−^* or *myh10^−/−^* single mutant males, which differs from what has been previously observed in mice (Yang *et al*. 2012) and suggests redundant roles between the *myh9b* (NMIIA) and *myh10* (NMIIB) in male zebrafish fertility.

Both *myh9a^−/−^* and *myh10^−/−^* single mutants presented with no phenotypic abnormalities or survival defects (Figs. 3 and 4); although, the only *myh9a;myh10* double mutant genotype that was viable as an adult was *myh9a^+/−^*; *myh10^+/−^*. We never identified any adults with the following genotypes: *myh9a^−/−^*; *myh10^−/−^*, *myh9a^+/−^*; *myh10^−/−^*, or *myh9a^−/−^*; *myh10^+/−^*. However, we did observe some common phenotypes in the progeny from the double heterozygous cross (*myh9a^+/−^*; *myh10^+/−^* X *myh9a^+/−^*; *myh10^+/−^*) which included: small size, curved tails, heart edema, and yolk edema (Fig. 7c and Table 2) (additional phenotype images can be found in Supplementary Fig. S5). These phenotypes indicate haploinsufficiency in the *myh9a*;*myh10* double mutant population. At 72 hpf, zebrafish larvae presenting with phenotypes were genotyped and identified as *myh9a^+/−^*; *myh10^+/−^* mutants, in contrast to the expected *myh9a^−/−^*; *myh10^−/−^* mutants. Next, we conducted a survival study in attempts to identify the time of death for any genotype other than *myh9a^+/−^*; *myh10^+/−^* (Supplementary Fig. S6); however, we were still unable to identify any fish with the following genotype combinations: *myh9a^−/−^*; *myh10^−/−^*; *myh9a^+/−^*; *myh10^−/−^*; or *myh9a^−/−^*; *myh10^+/−^*. Zebrafish progeny followed through the survival study had decreased survival rates compared to WT, but did not have decreased survival rates compared to their single *myh9a^−/−^* and *myh10^−/−^* mutant counterparts (Supplementary Fig. S6). The *myh9a;myh10* mutant combination revealed that one functional allele within either genotype was not sufficient to maintain normal function. Given these results, we predict that the *myh9a* and *myh10* genes have significant redundant functions that are masked in single mutants or double heterozygous animals. Additional experiments are needed to determine the cause of death or failure to produce the listed genotypes.

Together, double mutant populations examined developed more severe phenotypes and phenotypes at earlier time points than their single mutant counterparts (Fig. 7a-c), indicating genetic interactions. Attempts were also made to generate a triple *myh9a;myh9b;myh10* mutant. However, we were only able to identify triple heterozygous mutants: *myh9a^+/−^; myh9b^+/−^; myh10^+/−^* and while they were not used for these studies, they appeared normal as adults. These results again indicate the presence of redundant roles for the NMII encoding *myh* genes in development and haploinsufficient genotypes.

### Summary of *myh9* and *myh10* gene interactions

Using our zebrafish models, we found that *myh9b* is the critical NMIIA encoding *myh* gene, as *myh9b* zebrafish mutants have a partially penetrant lethal phenotype and develop pericardial edema. In zebrafish, the development of pericardial edema has been shown to result from a variety of causes including, but not limited to: impaired ciliogenesis, kidney defects, abnormal heart development, defects in vascularization, and defects in angiogenesis (Hentschel *et al*. 2005; Kramer-Zucker *et al*. 2005; Cianciolo Cosentino *et al*. 2010; Mitchell *et al*. 2010; Ben Shoham *et al*. 2012; Hegarty *et al*. 2013; Kumar *et al*. 2018; Yue *et al*. 2018; Luo *et al*. 2021; Huang *et al*. 2022). Although it was not examined here, we hypothesize the pericardial edema in the *myh9b* mutants is likely related to defects in kidney development based on the known role for *MYH9* in human kidney disease.

**Fig. 8.**
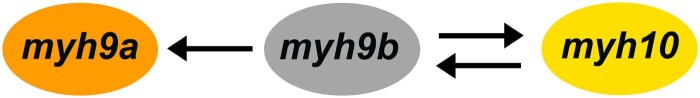
Summary of genetic interactions found through the analysis of the zebrafish *myh* mutant lines. *myh9b* is required for the expression of *myh9a* and *myh10*. *myh10* is required for the expression of *myh9b*.

Examination of gene expression by qRT-PCR did not reveal compensation by the *myh9a*, *myh9b*, or *myh10* genes, but we found these genes influence each other, with *myh9b* required for the expression of *myh9a* and *myh10*, and *myh10* required for the expression of *myh9b* (Fig. 8). However, establishing the mechanisms by which these genes were required for the expression of one another was out of the scope of this study. In zebrafish, it has been demonstrated that the degradation of mutant mRNA can trigger transcriptional adaptation events in which the expression of related genes can be upregulated (El-Brolosy *et al*. 2019). This work is interesting as it highlights a potential mechanism in which mutant mRNA could regulate the expression of other genes. While we observed decreases in gene expression in our mutants, rather than increases, we cannot rule out the possibility of a small mRNA or protein fragment being generated in our mutants. If a mutant fragment is produced it could potentially function in an unknown manner to alter the expression of other genes. Investigating these mechanisms in the future will be important in understanding genetic disease. We also identified alterations in protein levels that did not coincide with observed changes in gene expression and may indicate an alternate, and unconventional, compensatory mechanism when one *myh* gene is lost. Therefore, future studies can address potential mechanisms that might lead to protein stability within *myh* mutants.

## Discussion

### *MYH9* and *MYH10* in human disease and mammalian model systems

In humans, there are a variety of diseases and clinical phenotypes resulting from mutations in the *MYH9* and *MYH10* genes. Therefore, examining the NMII encoding *myh* genes is critical for understanding their roles in human development and disease. By establishing the first in vivo vertebrate *myh9a* (NMIIA), *myh9b* (NMIIA), and *myh10* (NMIIB) loss-of-function models we were able to investigate the role of these genes during embryonic and larval development. Mammalian loss-of-function models for *Myh9* and *Myh10* both resulted in lethality during early development (Tullio *et al*. 1997; Conti *et al*. 2004). However, there has been some success in the generation of mouse models with common patient specific mutations in *Myh9* at the R702, D1424, and E1841 residues, and in *Myh10* at the conserved R709 residue (Ma *et al*. 2004; Zhang *et al*. 2012; Suzuki *et al*. 2013). *Myh9* mutants recapitulate similar disease phenotypes found in humans with *MYH9*-related diseases as all develop hematologic defects, and some develop kidney abnormalities, cataracts, and hearing loss, which have allowed for critical advances in the understanding of genotype-phenotype connections (Zhang *et al*. 2012; Suzuki *et al*. 2013). *Myh10* mutants at the R709 residue displayed defects in neuronal migration and an inability to maintain proper balance (Ma *et al*. 2004). Together, the ablation mutants and the mutation specific models highlight the critical and unique functions of the NMIIA and NMIIB proteins.

Patient specific mutations in the *Myh9* and *Myh10* genes also present challenges in studying early development. Access to the tissue specific regions affected in disease using mammalian systems is not feasible in utero and the complete loss-of-function mutant phenotypes do not allow for the investigation of disease mechanisms due to their lethality. Therefore, the loss-of-function *myh9a*, *myh9b*, and *myh10* zebrafish mutants established in this study provide critical and accessible model systems for in vivo studies of the NMII encoding genes, their unique functions, and their critical interactions.

### Nonmammalian model systems for the study of NMII

Nonmammalian model systems such as *Caenorhabditis elegans* (*C*. *elegans*) and *Drosophila melanogaster* (*Drosophila*) have also been established to study the function of *MYH9* and *MYH10* in vivo. Many of these studies have focused on the discovery of cellular functions for these genes. *C*. *elegans* have three non-muscle myosin encoding genes (*nmy-1*, *nmy-2*, and *nmy-3*) (Johnson *et al*. 2022), which have been found to play roles in a variety of cellular processes including: the establishment of asymmetrical cell divisions to determine polarity in the embryo, the formation and maintenance of the cytokinesis furrow, and the specification of cell fate in both endodermal and seam cell lineages (Guo and Kemphues 1996; Liu *et al*. 2010; Ding and Woollard 2017). *Drosophila* studies have also revealed many critical roles for NMII in development and cellular function including: polarity, regulation of cell shape, migration of cardiac progenitor cells, organization of actin assemblies within the neuromuscular junction, force generation leading to the establishment of left–right tissue asymmetry, and implication in Notch signaling (Young *et al*. 1993; Okumura *et al*. 2010; Hunter *et al*. 2019; Doerflinger *et al*. 2022; Balaghi *et al*. 2023; Ermanoska and Rodal 2023).

Consistent with our observations in the zebrafish, *C*. *elegans nmy-1* and *nmy-2* genes have been shown to function redundantly (Piekny *et al*. 2003), although disease models for *MYH9* and *MYH10* using *C*. *elegans* have not been explored. In contrast, *Drosophila* studies are unable to add to the understanding of *myh* gene interactions as they express only one non-muscle myosin encoding gene, *zipper/*MyoII. Regardless, some *Drosophila* mutant models have been developed to explore “*MYH9*-like” mutations at conserved regions within their NMII heavy chain (R1171C, D1430N, D1847K, and R1939X) (Franke *et al*. 2007). These single amino acid mutants were recessive, hypomorphic, and displayed normal localization and self-assembly; however, when mutations were introduced into specific sensitized genetic backgrounds, they displayed dominant phenotypes (Franke *et al*. 2007). Therefore, while *Drosophila* mutants do not recapitulate the disease phenotypes of human patients with *MYH9*-RD, these animals provide important in vivo models systems for the potential identification of unknown *zipper*/MyoII genetic interactors (Franke *et al*. 2007). Identifying these interactors in *Drosophila* could be further explored in other model systems with multiple NMII encoding genes. Since *MYH9*-RD is an autosomal dominant condition, in which one mutant allele can result in disease development (Savoia and Pecci 2008), it is possible that interacting genes play critical roles in the presentation of the dominant phenotype. Therefore, it is feasible that individuals with one mutant allele, but without disease phenotypes, could be explained by presence of interacting genes or unknown interacting polymorphisms.

### Double mutant phenotypes and genetic redundancy

The analysis of zebrafish double mutants confirmed genetic interactions between the different NMII encoding genes, revealing both distinct and redundant roles for each of these genes throughout development. Consistent with our observations in the zebrafish double mutants, *C*. *elegans nmy-1* and *nmy-2* genes, which encode NMII isoforms, have also been shown to function redundantly during embryonic elongation (Piekny *et al*. 2003). Future studies in the *myh9a*;*myh10* double mutants at earlier time points would be needed to determine what processes these genes are required for throughout development. The double mutant analyses indicated that the *myh9a*, *myh9b*, and *myh10* genes likely share tissue specific redundant roles, and the zebrafish models developed in this study provide the tools required to further investigate their roles in development and disease.

### NMII haploinsufficiency in disease development

Haploinsufficiency occurs when the presence of one WT allele is not sufficient to maintain normal cellular function, resulting in disease phenotypes in heterozygous individuals. In human patients with *MYH9*-RD, macrothrombocytopenia is a common phenotype, which is characterized by abnormally large platelet populations, and a small number of circulating platelets (Savoia and Pecci 2008). Studies of patients with *MYH9*-RD containing R1933X and E1945X mutations revealed reduced levels of NMIIA in platelets and megakaryocytes (Pecci *et al*. 2005). Further, it was discovered that only WT NMIIA was expressed in these cells, suggesting that the reduced NMIIA expression, rather than a dominant-negative effect, likely contributed to these disease manifestations (Pecci *et al*. 2005). Therefore, in megakaryocytic lineages where NMIIA is the only isoform expressed, it appears as though haploinsufficiency is the primary cause of hematological manifestations.

In contrast, mouse models with the R702C, D1424N, and E1841K point mutations, simple haploinsufficiency was ruled out as the likely mechanism leading to disease phenotypes due to similarities in WT and mutant NMIIA protein levels (Zhang *et al*. 2012). Simple haploinsufficiency is also ruled out in our zebrafish *myh9a* and *myh10* mutants as neither heterozygous nor homozygous mutants display any phenotypes with complete loss of WT gene expression. However, while the pericardial edema phenotype in the *myh9b* mutants is largely associated with the *myh9b^−/−^* genotype, some *myh9b^+/−^* mutants also developed pericardial edema (data not shown). In cases where *myh9b^+/−^* mutants developed pericardial edema, we hypothesize it was due to haploinsufficiency. While pericardial edema is a similar phenotype among all double mutant lines, there were also a variety of genotype specific distinct phenotypes that developed, suggesting these genes likely have tissue specific redundant roles.

### Dominant-negative NMII interactions

*MYH9* and *MYH10* mutations may contribute to disease manifestations through a dominant-negative effect of the mutant allele. Dominant-negative interactions occur when a mutant allele is capable of interfering with the function of a WT allele. In *MYH9*-RD, granulocyte inclusions consist of cytoplasmic NMIIA accumulations (Savoia and Pecci 2008), which have been shown to contain WT NMIIA, suggesting that dominant-negative interactions may contribute to this disease phenotype (Pecci *et al*. 2005). Common and conserved mutations in the rod domain of NMIIA (R1165C, D1424N, E1841K, and R1933Stop) have also been shown to result in defects in NMIIA bipolar filament assembly in vitro (Franke *et al*. 2005). These mutations were found to either disrupt the folding of molecules into α-helical coiled-coil structures or disrupt the lateral associations among molecules required to form higher ordered assemblies (Franke *et al*. 2005), further supporting a dominant-negative mechanism. Additionally, overexpression of patient-specific *MYH10* in vitro resulted in shortened primary cilia, suggesting that some *MYH10* variants may have dominant-negative effects as well (Holtz *et al*. 2022). Finally, expression of motor-impaired NMIIB was capable of interfering with WT NMIIA function, highlighting a novel role for dominant-negative effects between different NMII isoforms (Ma and Adelstein 2014).

These findings are interesting as they pose important questions regarding how the NMII isoforms interact with one another and how NMII proteins interact to form homotypic or heterotypic filaments within cells. In our complete loss-of-function mutations, we do not expect dominant-negative mechanisms to interfere with the WT NMII proteins; however, we cannot rule out the possibility of a small and undetectable protein fragment being expressed that could interfere with normal protein function.

### NMII bipolar filament interactions

NMII proteins have been shown to interact through the formation of homotypic and heterotypic bipolar filaments, consisting of one isoform exclusively, or any combination of the three NMIIA, NMIIB, or NMIIC isoforms (Beach *et al*. 2015). Heterotypic filaments have been shown to be common in regions where both isoforms are expressed, but also present in regions where one is more predominantly expressed (Beach *et al*. 2015). While NMII bipolar filament formation has not yet been studied in zebrafish, the formation of heterotypic bipolar filaments in other model systems leads us to hypothesize that the zebrafish NMII isoforms are capable of homotypic or heterotypic assemblies. Our in situ hybridization data indicate the *myh9b* and *myh10* have largely overlapping expression profiles making it reasonable to hypothesize that heterotypic filament may occur in many tissue types within the developing embryo and larvae. Further, since zebrafish have two *myh9* genes, *myh9a* and *myh9b*, both of which produce NMIIA isoforms, future studies are needed need to explore whether the *myh9a* (NMIIA) and *myh9b* (NMIIA) can form heterotypic filaments and whether they co-localize or have distinct localizations and cellular functions.

## Conclusion

The zebrafish *myh9a*, *myh9b*, and *myh10* mutants outlined in this study provide valuable model systems to elucidate genetic interactions between the *myh9* and *myh10* genes. The most prominent single mutant phenotype was the development of pericardial edema in *myh9b* mutant zebrafish; however, the mechanisms underlying the cause of edema were not explored here. The known role of the NMIIA protein in the regulation of cell shape, cell migration, cellular contractility, and cell adhesion, suggest there is likely more than one biological process disrupted in the *myh9b* mutants (Even-Ram *et al*. 2007; Gutzman *et al*. 2015; Smith *et al*. 2018). The absence of phenotypes in the *myh9a* and *myh10* zebrafish mutants was interesting, as *Myh9* and *Myh10* loss-of-function mouse mutants present with severe and lethal phenotypes. These genes may be less critical in zebrafish development, or the lack of phenotypes may be due to alternative unknown compensatory mechanisms, which could allow for the phenotypic reversal in the *myh9b* mutants and the absence of phenotypes in the *myh9a* and *myh10* mutants.

Future quantification of cell-type specific NMII isoform expression will be crucial to understand potential genetic interactions, as regions in which one isoform is exclusively expressed or highly enriched compared to others, may elucidate cell-type specific disease manifestations. Furthermore, regions in which more than one isoform is highly expressed may provide information regarding genetic interactors or potential compensatory partners. While further work is required to determine the cause of the *myh9b* mutant pericardial edema phenotype and the exact mechanisms by which these genes interact with one another, our *myh9a*, *myh9b*, and *myh10* zebrafish mutants are an exciting development as they are the first model systems in which loss-of-function mutations in the NMIIA and NMIIB encoding *myh* genes can be explored in vivo throughout development.

## Supplementary Material

jkae260_Supplementary_Data

## Data Availability

Zebrafish strains and plasmids are available upon request. The authors affirm that all data necessary for confirming the conclusions of the article are present within the article, figures, and tables. Supplemental material available at G3 online.
