# Supplementary material for: myh9b is a critical non-muscle myosin II encoding gene that interacts with myh9a and myh10 during zebrafish development in both compensatory and redundant pathways
Source: G3 (Bethesda). 2024 Nov 6;15(1):jkae260. doi: 10.1093/g3journal/jkae260 (PMC11708221; doi:10.1093/g3journal/jkae260)
Supplement: jkae260_Supplementary_Data [file jkae260_supplementary_data.zip › Figure_S2_G3-2024-405427.docx]

**Figure S2.**


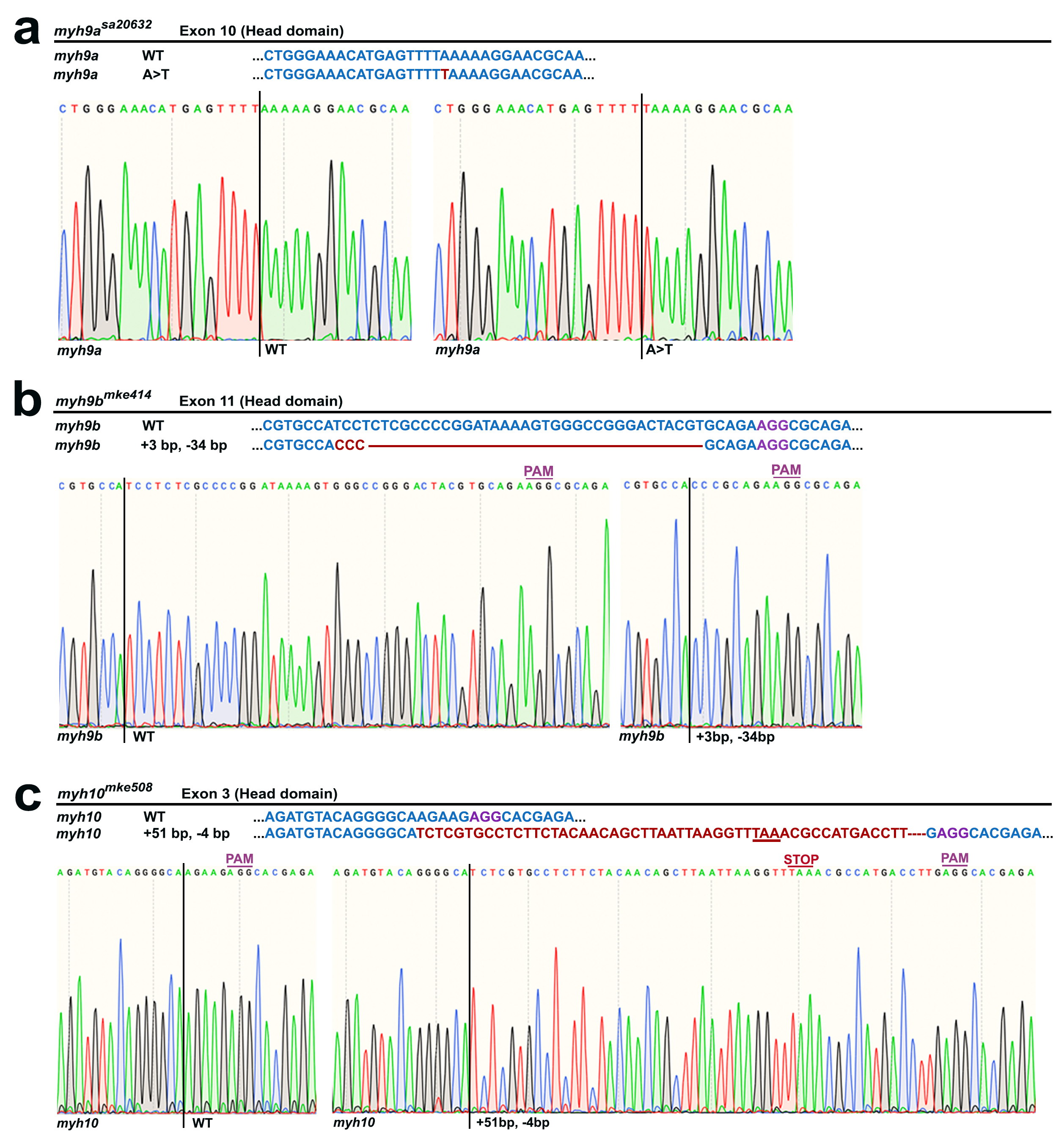


**Figure S2. Trace files for *myh* mutants used in these studies showing wild-type and mutant sequences. Wild-type sequence in blue and mutant sequence in red.** a) *myh9a* mutant obtained from the Sanger Zebrafish Mutation Project. b) *myh9b* mutant generated for this study with PAM indicated in purple. c) *myh10* mutant generated for this study with in-frame stop codon indicated in red and PAM in purple.
