# Supplementary material for: myh9b is a critical non-muscle myosin II encoding gene that interacts with myh9a and myh10 during zebrafish development in both compensatory and redundant pathways
Source: G3 (Bethesda). 2024 Nov 6;15(1):jkae260. doi: 10.1093/g3journal/jkae260 (PMC11708221; doi:10.1093/g3journal/jkae260)
Supplement: jkae260_Supplementary_Data [file jkae260_supplementary_data.zip › Figure_S3_G3-2024-405427.docx]

**Figure S3.**


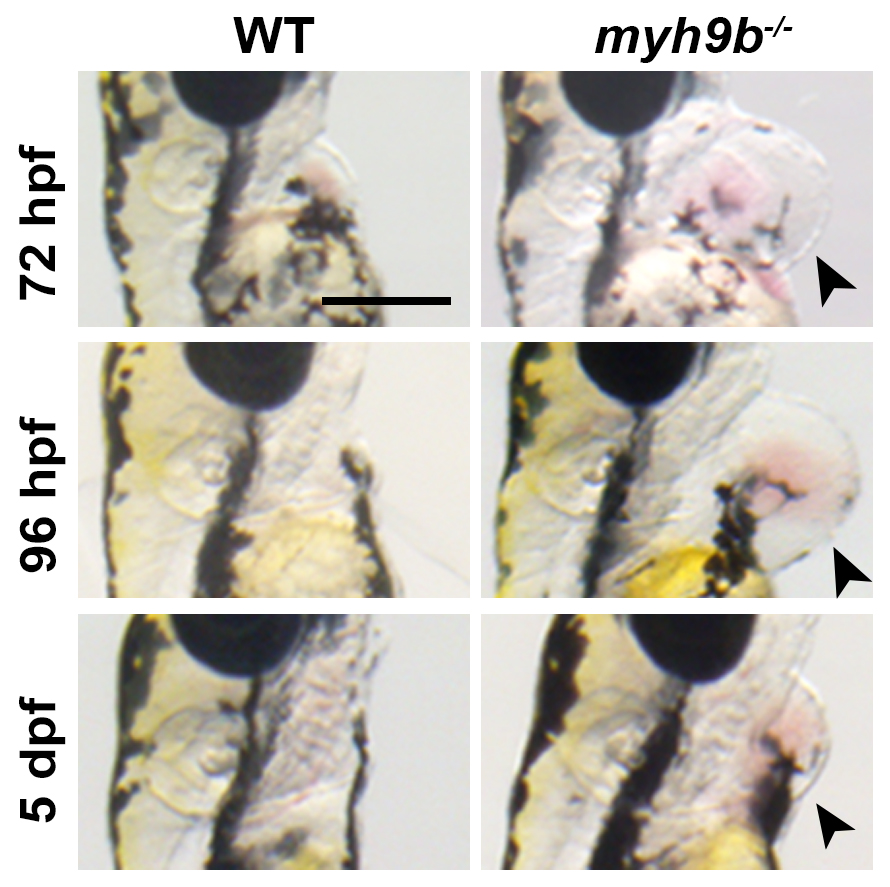


**Figure S3. Wild-type and *myh9b^-/-^* mutants imaged from Figure 3 showing close-up of heart edema severity.** Arrowheads indicate pericardial edema at 72 hpf and 96 hpf, and phenotype reversal at 5 dpf. Scale = 0.25 mm.
