# Supplementary material for: myh9b is a critical non-muscle myosin II encoding gene that interacts with myh9a and myh10 during zebrafish development in both compensatory and redundant pathways
Source: G3 (Bethesda). 2024 Nov 6;15(1):jkae260. doi: 10.1093/g3journal/jkae260 (PMC11708221; doi:10.1093/g3journal/jkae260)
Supplement: jkae260_Supplementary_Data [file jkae260_supplementary_data.zip › Figure_S4_G3-2024-405427.docx]

**Figure S4.**


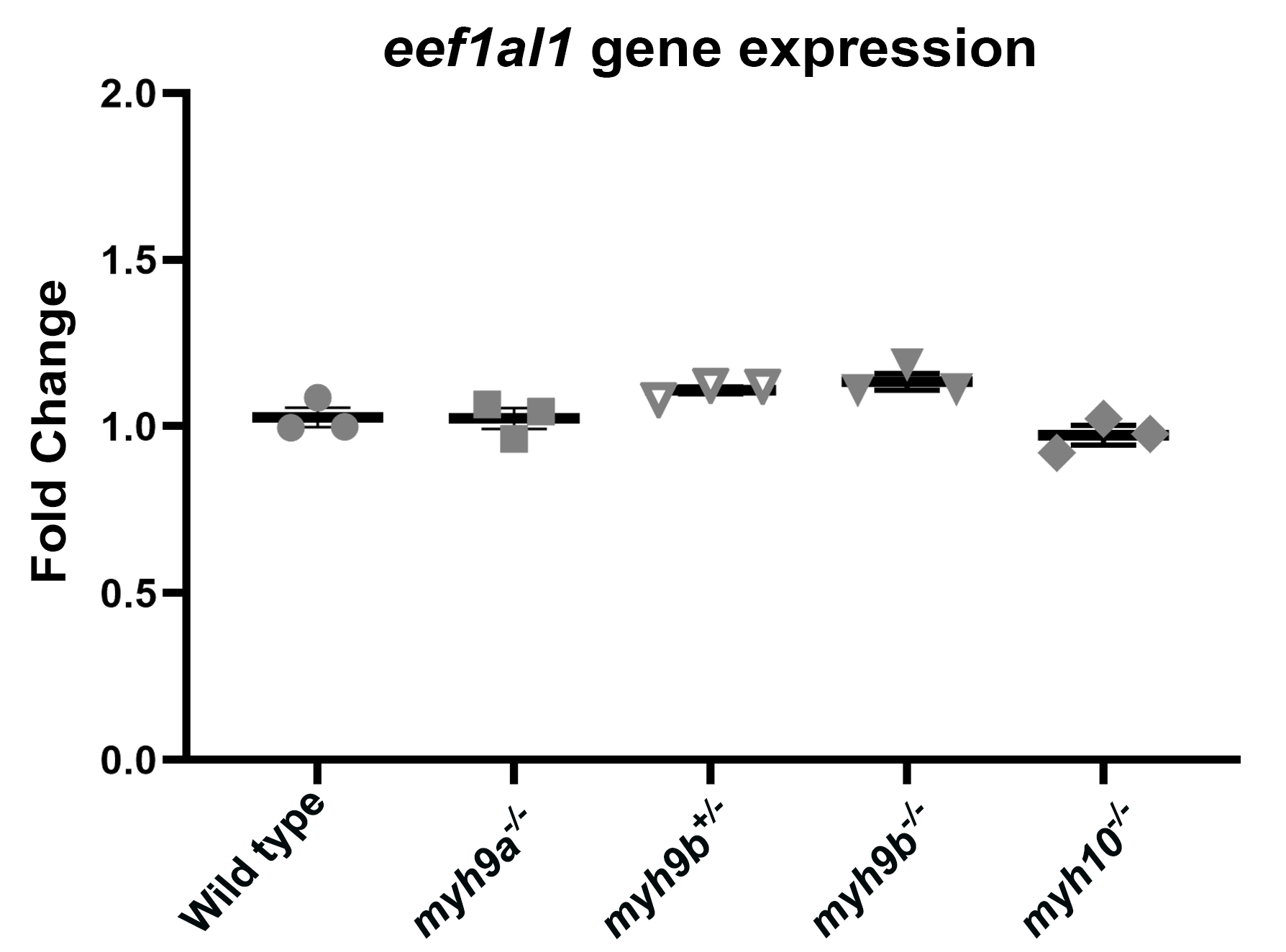


**Figure S4. Control qRT-PCR for *eef1al1* gene expression in each larval genotype at 96 hpf.** Primer sequences can be found in Table S5.
