# Supplementary material for: myh9b is a critical non-muscle myosin II encoding gene that interacts with myh9a and myh10 during zebrafish development in both compensatory and redundant pathways
Source: G3 (Bethesda). 2024 Nov 6;15(1):jkae260. doi: 10.1093/g3journal/jkae260 (PMC11708221; doi:10.1093/g3journal/jkae260)
Supplement: jkae260_Supplementary_Data [file jkae260_supplementary_data.zip › Figure_S5_G3-2024-405427.docx]

**Figure S5.**


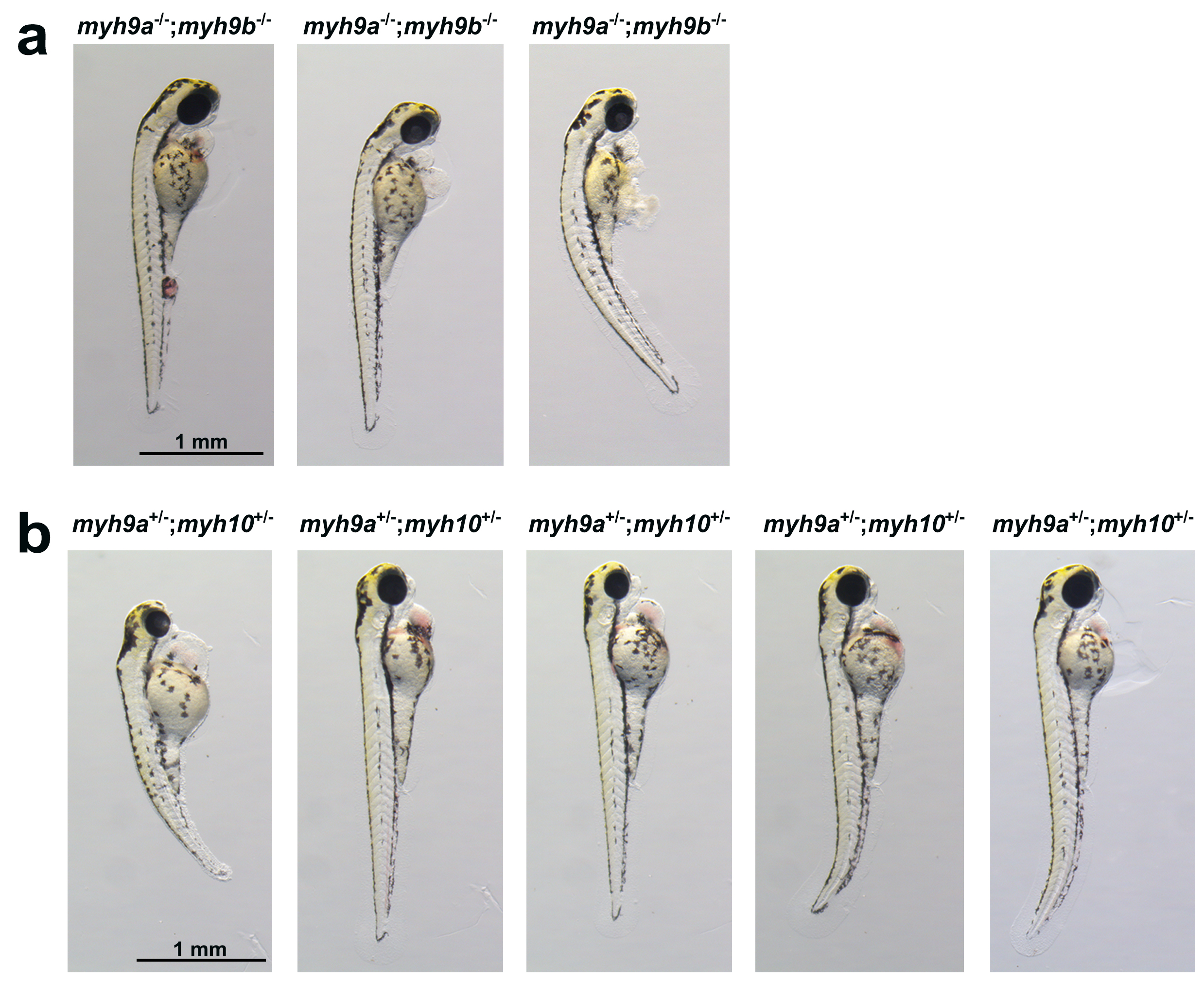


**Figure S5. Additional *myh* double mutant phenotype images.** a) *myh9a^-/-^;myh9b^-/-^* larvae at 72 hpf showing heart edema, tail cysts, and skin blistering. b) *myh9a^+/-^;myh10^+/-^* larvae at 72 hpf showing heart edema, curved body axes, and abnormal jaw development. Scale bars = 1 mm for all images.
