# Supplementary material for: myh9b is a critical non-muscle myosin II encoding gene that interacts with myh9a and myh10 during zebrafish development in both compensatory and redundant pathways
Source: G3 (Bethesda). 2024 Nov 6;15(1):jkae260. doi: 10.1093/g3journal/jkae260 (PMC11708221; doi:10.1093/g3journal/jkae260)
Supplement: jkae260_Supplementary_Data [file jkae260_supplementary_data.zip › Figure_S6_G3-2024-405427.docx]

**Figure S6.**


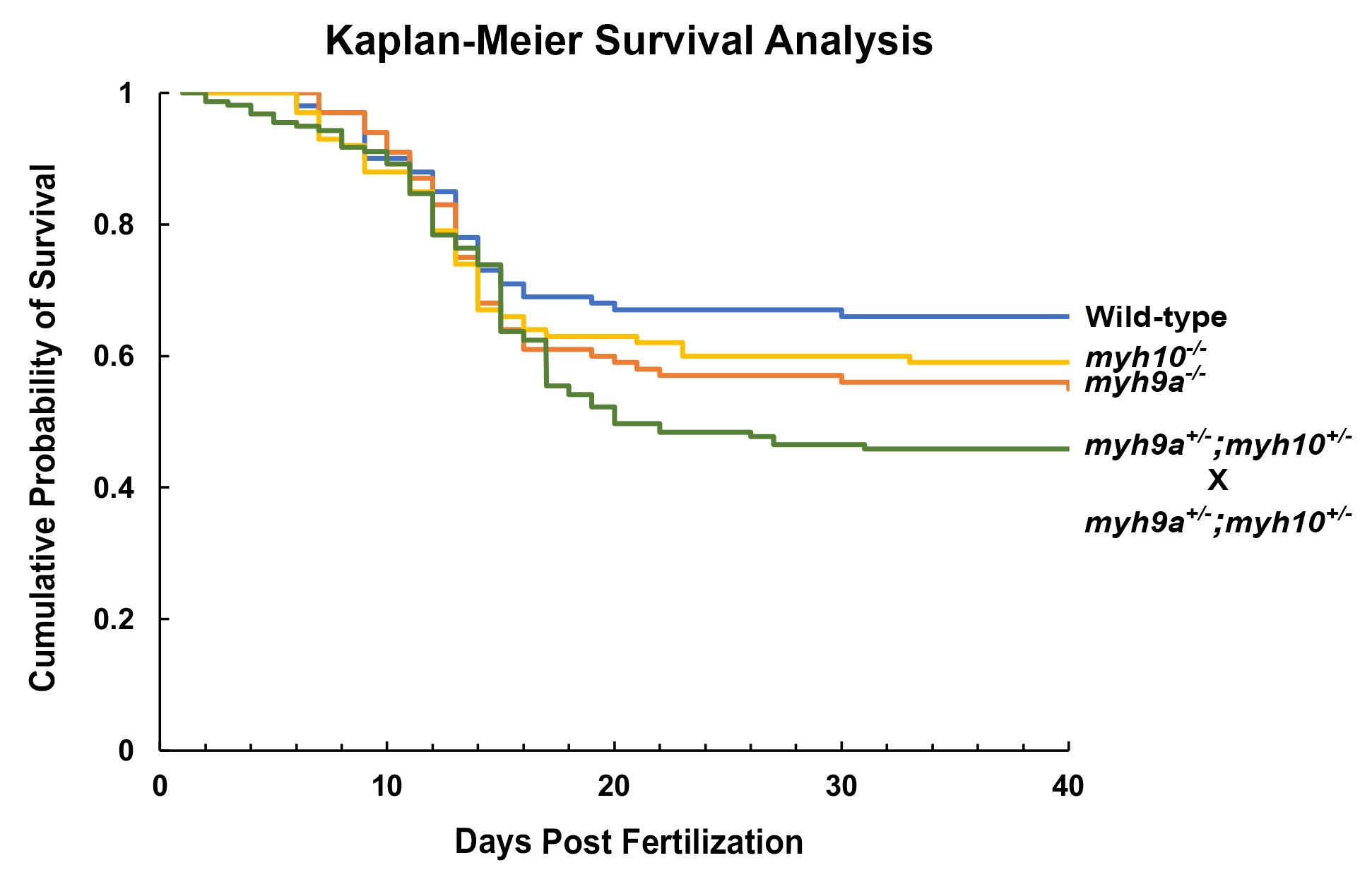


**Figure S6. Kaplan-Meier survival curve for progeny produced from *myh9a^+/-^;myh10^+/-^* X *myh9a^+/-^;myh10^+/-^* cross.** The *myh9a;myh10* double mutant population has decreased survival rates compared to wild type (log-rank tests P < 0.01) but not to *myh9a^-/-^* or *myh10^-/-^* single mutants. Wild-type, n=100. *myh9a^-/-^*, n=100. *myh10^-/-^*, n=100. *myh9a;myh10*, n= 157.
