# Supplementary material for: myh9b is a critical non-muscle myosin II encoding gene that interacts with myh9a and myh10 during zebrafish development in both compensatory and redundant pathways
Source: G3 (Bethesda). 2024 Nov 6;15(1):jkae260. doi: 10.1093/g3journal/jkae260 (PMC11708221; doi:10.1093/g3journal/jkae260)
Supplement: jkae260_Supplementary_Data [file jkae260_supplementary_data.zip › Table_S2_G3-2024-405427.docx]

**Table S2. Transcript ID’s for zebrafish and human genes used in these studies**

| **Organism** | **Gene** | **Ensembl ID** | **Transcript** | **Ensembl transcript ID** |
| --- | --- | --- | --- | --- |
| Human | *MYH9* | ENST00000216181.11 | 201 | ENST00000216181.11 |
| Human | *MYH10* | ENST00000360416.8 | 202 | ENST00000360416.8 |
| Human | *MYH14* | ENST00000642316.2 | 208 | ENST00000642316.2 |
| Zebrafish | *myh9a* | ENSDARG00000063295 | 202 | ENSDART00000149823.2 |
| Zebrafish | *myh9b* | ENSDARG00000001014 | 202 | ENSDART00000137105.3 |
| Zebrafish | *myh10* | ENSDARG00000000103 | 202 | ENSDART00000151114.2 |
| Zebrafish | *myh14* | ENSDARG00000073732 | 203 | ENSDART00000142155.3 |
