# Supplementary material for: myh9b is a critical non-muscle myosin II encoding gene that interacts with myh9a and myh10 during zebrafish development in both compensatory and redundant pathways
Source: G3 (Bethesda). 2024 Nov 6;15(1):jkae260. doi: 10.1093/g3journal/jkae260 (PMC11708221; doi:10.1093/g3journal/jkae260)
Supplement: jkae260_Supplementary_Data [file jkae260_supplementary_data.zip › Table_S3_G3-2024-405427.docx]

**Table S3. Mutant generation target sequence information**

| ***myh* mutant line** | **gRNA target sequence information**  **with PAM (5’->3’)** | **Gene specific oligonucleotide (lowercase) flanked by target sequence information (uppercase)** | **Constant oligonucleotide** |
| --- | --- | --- | --- |
| *myh9b^mke414^* | GGGCCGGGACTACGTGCAGAAGG (exon 11) | TAATACGACTCACTATA  gggccgggactacgtgcaga GTTTTAGAGCTAGAAATAGCAAG | AAAAGCACCGACTCGGTGCCACTTTTTCAAGTTGATAACGGACTAGCCTTATTTTAACTTGCTATTTCTAGCTCTAAAAC |
| *myh10^mke508^* | AGATGTACAGGGGCAAGAAGAGG (exon 3) | TAATACGACTCACTATA  agatgtacaggggcaagaag  GTTTTAGAGCTAGAAATAGCAAG |  |
