# Supplementary material for: myh9b is a critical non-muscle myosin II encoding gene that interacts with myh9a and myh10 during zebrafish development in both compensatory and redundant pathways
Source: G3 (Bethesda). 2024 Nov 6;15(1):jkae260. doi: 10.1093/g3journal/jkae260 (PMC11708221; doi:10.1093/g3journal/jkae260)
Supplement: jkae260_Supplementary_Data [file jkae260_supplementary_data.zip › Table_S4_G3-2024-405427.docx]

**Table S4. HRMA primers used for *myh9b* and *myh10* mutant generation**

| **Gene** | **Forward Primer (5’->3’)** | **Reverse Primer (5’->3’)** |
| --- | --- | --- |
| *myh9b* | CTGATGGGCATGAATGTGAC | AACATCCTC TCGTATGTGGCTT |
| *myh10* | TAAACCCATACAAAAACCTCCC | AAATGTGGTCTTACCTTGAAGCA |
