# Supplementary material for: myh9b is a critical non-muscle myosin II encoding gene that interacts with myh9a and myh10 during zebrafish development in both compensatory and redundant pathways
Source: G3 (Bethesda). 2024 Nov 6;15(1):jkae260. doi: 10.1093/g3journal/jkae260 (PMC11708221; doi:10.1093/g3journal/jkae260)
Supplement: jkae260_Supplementary_Data [file jkae260_supplementary_data.zip › Table_S6_G3-2024-405427.docx]

**Table S6. Wholemount *in situ* hybridization total sample numbers within each timepoint analyzed**

| **Timepoint and Gene** | **Antisense** | **Sense** |
| --- | --- | --- |
| 48 hpf *myh9a* | 37 | 5 |
| 48 hpf *myh9b* | 39 | 6 |
| 48 hpf *myh10* | 20 | 4 |
| 72 hpf *myh9a* | 50 | 16 |
| 72 hpf *myh9b* | 29 | 8 |
| 72 hpf *myh10* | 42 | 18 |
| 96 hpf *myh9a* | 78 | 14 |
| 96 hpf *myh9b* | 83 | 13 |
| 96 hpf *myh10* | 73 | 18 |
